# The benefits of smoking cessation on survival in cancer patients by integrative analysis of multi‐omics data

**DOI:** 10.1002/1878-0261.12755

**Published:** 2020-07-11

**Authors:** Sheng Yang, Tong Liu, Geyu Liang

**Affiliations:** ^1^ Key Laboratory of Environmental Medicine Engineering Ministry of Education School of Public Health Southeast University Nanjing China

**Keywords:** current smokers, prognosis, smoking cessation, smoking signature, true effect

## Abstract

Few studies have examined the association between smoking status (including former smokers) at diagnosis and overall survival among cancer patients. We aimed to assess the benefits of quitting smoking on cancer prognosis in cohorts of cancer patient smokers obtained from the Cancer Genome Atlas (TCGA) database. Hazard ratios (HR) were calculated to evaluate smoking behavior at cancer diagnosis (reformed smokers vs. current smokers) in association with overall survival using multivariate‐adjusted Cox regressions analysis. According to our analyses, quitting smoking was the independent protective factor for overall survival in lung squamous cell carcinoma (LUSC) (HR = 0.67, 95% CI = 0.48–0.94). Comprehensive analysis of multicomponent data across reformed and current smokers identified a total of 85 differential expressed genes (DEGs) affected by different modes of genetic and epigenetic regulation, potentially representing cancer drivers in smokers. Moreover, we provided a smoking‐associated gene expression signature, which could evaluate the true effect on prognosis with high power (HR = 1.70, 95% CI = 1.19–2.43, AUC = 0.65, 0.67, and 0.70 for 2‐, 3‐, and 5‐year survival, respectively). This signature was also applicable in other smoking‐related cancers, including bladder urothelial carcinoma (HR = 1.70, 95% CI = 1.01–2.88), cervical carcinoma (HR = 5.69, 95% CI = 1.37–23.69), head and neck squamous cell carcinoma (HR = 1.97, 95% CI = 1.41–2.76), lung adenocarcinoma (HR = 1.73, 95% CI = 1.16–2.57), and pancreatic adenocarcinoma (HR = 4.28, 95% CI = 1.47–12.47). In conclusion, this study demonstrates that quitting smoking at diagnosis decreases risk of death in cancer patients. We also provide a smoking‐associated gene expression signature to evaluate the effect of smoking on survival. Lastly, we suggest that smoking cessation could comprise a part of cancer treatment to improve survival rates of cancer patients.

## Graphical abstract

Tobacco smoking is an established risk factor for many cancers' development. In the present study, we used the the Cancer Genome Atlas cohort to estimate the association between smoking cessation and overall survival, understand the genetic and immune microenvironment of smoking patients, and provide an effective smoking signature for evaluating the smoking level to predict prognosis.

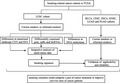

**Abbreviations**
BLCA: bladder urothelial carcinoma
ceRNA: the competing endogenous RNAs
CESC: cervical squamous cell carcinoma and endocervical adenocarcinoma
CIBERSORT: Cell‐type Identification by Estimating Relative Subsets of RNA Transcripts
CIs: confidence intervals
CNV: copy number variation
DEGs: differential expressed genes
ESCA: esophageal carcinoma
GO: Gene Ontology
HNSC: head and neck squamous cell carcinoma
HPV: human papillomavirus
HR: hazard ratios
KEGG: Kyoto Encyclopedia of Genes and Genomes pathway
LUAD: lung adenocarcinoma
LUSC: lung squamous cell carcinoma
PAAD: pancreatic adenocarcinoma
ROC: receiver operating characteristic curves
TCGA: the Cancer Genome Atlas
TIMER: the Tumor IMmune Estimation Resource

## 1 Introduction

Tobacco smoking is a risk factor for the occurrence and increases the incidence of various cancers, including bladder [1], head and neck [2], lung [3], and pancreatic cancer [4]. Tobacco smoking contains many carcinogenic chemicals that can create a specific mutational signature and increase the somatic mutational burden associated with unrepaired DNA damage [5]. In addition to causing frequent gene mutations, tobacco smoking also appears to break the immune homeostasis, which may contribute to tumorigenesis [6]. These effects of smoking on the immune system and genetic materials can be considered as the true effect of smoking. It is recognized that continuous smoking not only has unhealthy impacts on the general population, but also is the negative prognostic factor for cancer patients by comparing smokers with never smokers in most studies [7, 8]. However, few studies have researched the association between smoking status changes (especially quitting smoking) and mortality among cancer patients compared with the general population. A study found that current smoking increased overall mortality risk compared with former smokers using multivariate Cox regression analysis [9]. Another study based on the Shanghai Cohort Study also found that a statistically significant increased mortality risk was associated with smoking relative to nonsmoking after cancer diagnosis [10]. A cohort in Japan also found that quitters had consistently higher survival rates than current smokers during a 10‐year calendar period after diagnosis among cancer patients and suggested that smoking cessation should be a part of cancer care [11]. These studies were based on a large population and adjusted for age, gender, stage, and other basic characters. However, some important prognostic factors were not considered, including human papillomavirus (HPV) status associated with the prognosis of head and neck cancer and cervical cancer [12], and tumor status after surgery associated with the prognosis in many cancers [13, 14]. It is another limit that the underlying mechanism of smoking cessation to improve survival time has not been further studied. Therefore, to understand the benefits of quitting smoking on prognosis among cancer patients, we first evaluated smoking status at cancer diagnosis (reformed smokers vs. current smokers) in association with overall survival and then comprehensively analyzed the transcriptome data, mutational profile, and immune microenvironment of smoking‐related cancers from the Cancer Genome Atlas (TCGA) database.

## 2 Materials and methods

### 2.1 Data source

This study used public data from the TCGA database. The information of smoking status, survival time, and the clinical characteristics was downloaded for bladder urothelial carcinoma (BLCA), cervical squamous cell carcinoma and endocervical adenocarcinoma (CESC), esophageal carcinoma (ESCA), head and neck squamous cell carcinoma (HNSC), lung adenocarcinoma (LUAD), lung squamous cell carcinoma (LUSC), and pancreatic adenocarcinoma (PAAD) from TCGA data portal (https://portal.gdc.cancer.gov/). Detailed patient characteristics of each cancer are given in Table S1. Besides, the gene expression RNAseq (HTSeq‐FPKM), miRNA expression RNAseq (Illumina HiSeq), somatic mutation data (SNV, VarScan2 Variant Aggregation, and Masking), copy number variation data (CNV, Masked Copy Number Segment hg38), and DNA methylation data (Illumina Human Methylation 450) of above cancers were also obtained from the TCGA database. Because the data were extracted from the TCGA database, following the publication guidelines strictly approved by TCGA, there was no requirement for ethics committee approval.

### 2.2 The association between smoking cessation and overall survival

The smoking status was included current smokers (included daily smokers and nondaily smokers or occasional smokers) and current reformed smokers (people who were not smoking at the time of the interview but have smoked at least 100 cigarettes in their life). To understand the association between smoking cessation and patients' overall survival, age and multivariate‐adjusted Cox regressions were performed to calculate the hazard ratio (HR) with 95% confidence intervals (CIs). In the multivariate model, we adjusted for age, gender, tumor stage, tumor status, and HPV status.

### 2.3 Differentially expressed gene analysis

Differentially expressed mRNAs (DEGs), lncRNAs, and miRNAs were identified between current smokers and reformed smokers (*P*‐value < 0.05) by ‘limma’ package with R [15]. Function analysis including Gene Ontology (GO) and Kyoto Encyclopedia of Genes and Genomes (KEGG) pathway was performed by ‘clusterProfiler’ package in R [16].

### 2.4 Somatic mutation analysis

The somatic mutation frequency ≥ 19 was considered to compare their relative distribution between current smokers and reformed smokers. Waterfall map for somatic mutation patterns was performed by the R package ‘GenVisR’ [17]. Then, the association between gene expression and somatic mutation was determined by the Mann–Whitney *U*‐test. The total mutation loads of current smokers and reformed smokers were compared using the Mann–Whitney *U*‐test.

### 2.5 Copy number variation analysis

Values of segment mean bigger than 0.2 were defined as gain and < −0.2 as loss [18]. Chi‐square test was used to compare CNV between current smokers and reformed smokers. Circos plots were performed by the R package ‘Rciorcos’ [19]. Then, the association between gene expression and CNV was determined by the Kruskal–Wallis test.

### 2.6 DNA methylation analysis

The gene methylation matrix was normalized by ‘limma’ package with R. The gene with different DNA methylation level between current smokers and reformed smokers was also used by ‘limma’ package. Then, the association between gene expression and DNA methylation level was determined by the Pearson correlation coefficient. Above differentially expressed genes (DEGs) related to SNV, CNV, or DNA methylation were considered as the key DEGs.

### 2.7 Construction of competing endogenous RNA network

The differentially expressed lncRNAs, miRNAs, and key DEGs were used to construct the competing endogenous RNA (ceRNA) network. LncRNA‐miR links were predicted by miRcode database. The targets of miRNAs were predicted by miRDB 6.0, mirTarBase 7.0, and TargetScan 7.2. The ceRNA network was visualized by Cytoscape 3.6 [20].

### 2.8 Immune cell scores

The abundance of immune cells was measured using three different algorithms, including the Tumor IMmune Estimation Resource (TIMER, six immune cell types) [21], Cell‐type Identification by Estimating Relative Subsets of RNA Transcripts (CIBERSORT, 22 immune cell types) [22], and xCell (64 immune and stromal cell types) [22]. The comparison of immune cell distribution between current smokers and reformed smokers was made using the Mann–Whitney *U*‐test.

### 2.9 Construction and validation of a smoking signature

To evaluate the true degree of smoking and predict the overall survival of smokers, we provided a quantitative smoking signature using key DEGs and immune cells. According to the somatic mutation, genes were valued as 0 (wild) and 1 (mutation). According to the CNV, genes were valued as −1(loss), 0 (normal), and 1 (gain). The immune cell fraction level was valued as 0 or 1. When the score of a type of cell exceeds the corresponding cut‐off value, it is assigned as 1; otherwise, it is assigned as 0. Smoking status was also important and included in the smoking signature (current smoking = 1; stopping smoking = 0). First, prognostic factors were identified by performing univariate Cox regression analysis. LASSO‐COX analysis was then employed to obtain the most useful predictive features. The smoking signature was built based on the corresponding coefficients.

The Kaplan–Meier (K‐M) survival curves and time‐dependent receiver operating characteristic [survival receiver operating characteristic curves (ROC)] curves were applied to evaluate the predicting ability of smoking signature using the ‘survivalROC’ package in R. We built a prognostic nomogram on the basis of the smoking signature and clinical information in smokers with LUSC using the ‘rms’ package in R. Calibration curves for 2, 3, and 5 years were also plotted to compare the predicted and actual probabilities.

We calculated the smoking signature in different cancers in the TCGA database using the same formula. To confirm the applicability and reliability of the smoking signature, age and multivariate‐adjusted Cox regressions were performed to calculate the HR with 95% CIs. In the multivariate model, we adjusted for age, gender, tumor stage, tumor status, and HPV status.

### 2.10 Statistics

All data were expressed as mean ± SD. LASSO‐COX analysis was performed using the ‘glmnet’ package. The optimal cut‐off of each immune cell type was assessed based on survival information and cell fraction using the ‘survminer’ package. Survival analysis used Cox proportional hazards model and K‐M curves. The above analysis was conducted using r software 3.5 (R Project for Statistical Computing, Vienna, Austria) and spss software 23.0 (IBM Corporation, Armonk, NY), and all statistical tests were bilateral, *P* < 0.05 was statistically significant.

## 3 Results

### 3.1 Quitting smoking can significantly improve the prognosis of cancer patients

The age and multivariable‐adjusted HRs for the association between smoking cessation and patients' overall survival are presented in Table 1. In age‐adjusted models, quitting smoking was significantly associated with longer survival time in CESC, HNSC, LUSC, and PAAD. In the multivariable‐adjusted model, though there was no significance in the majority of cancers, reformed smokers had a better prognosis than current smokes. Only in LUSC, the multivariable‐adjusted HR (95% CI) was 0.67 (0.48–0.94) among reformed smokers relative to current smokers, indicating that quitting smoking was the independent protective factor for prognosis. Therefore, we further explored the potential mechanism by which quitting smoking can improve the prognosis in LUSC.

**Table 1:** The association between smoking status and patients' overall survival.

Cancer type	Current smoker	Reformed smoker	Age‐adjusted HR (95% CI)	*P*‐value	MV‐adjusted HR (95% CI) ^a^	*P*‐value
BLCA	91	198	0.79 (0.54–1.16)	0.230	0.72 (0.42–1.22)	0.219
CESC ^b^	64	53	**0.34 (0.16**–**0.71)**	**0.004**	0.63 (0.26–1.53)	0.309
ESCA	37	73	0.89 (0.46–1.72)	0.723	0.89 (0.39–2.02)	0.780
HNSC ^b^	178	215	**0.65 (0.47**–**0.89)**	**0.007**	0.67 (0.44–1.02)	0.059
LUAD	122	311	1.12 (0.77–1.63)	0.561	1.34 (0.85–2.10)	0.207
LUSC	134	340	**0.62 (0.46**–**0.83)**	**0.001**	**0.67 (0.48**–**0.94)**	**0.020**
PAAD	20	60	**0.45 (0.23**–**0.89)**	**0.021**	0.54 (0.24–1.20)	0.132

Significant associations are shown in bold.

^a^ MV‐adjusted for age (continuous), gender (female, male), tumor stage (stage Ⅰ, stage Ⅱ, stage Ⅲ, and stage Ⅳ), and tumor status (with tumor and tumor‐free).

^b^ MV‐adjusted for age (continuous), gender (female, male), tumor stage (stage Ⅰ, stage Ⅱ, stage Ⅲ, and stage Ⅳ), tumor status (with tumor and tumor‐free), and HPV status (positive and negative).

### 3.2 Differentially expressed gene analysis

**Fig. 1:**
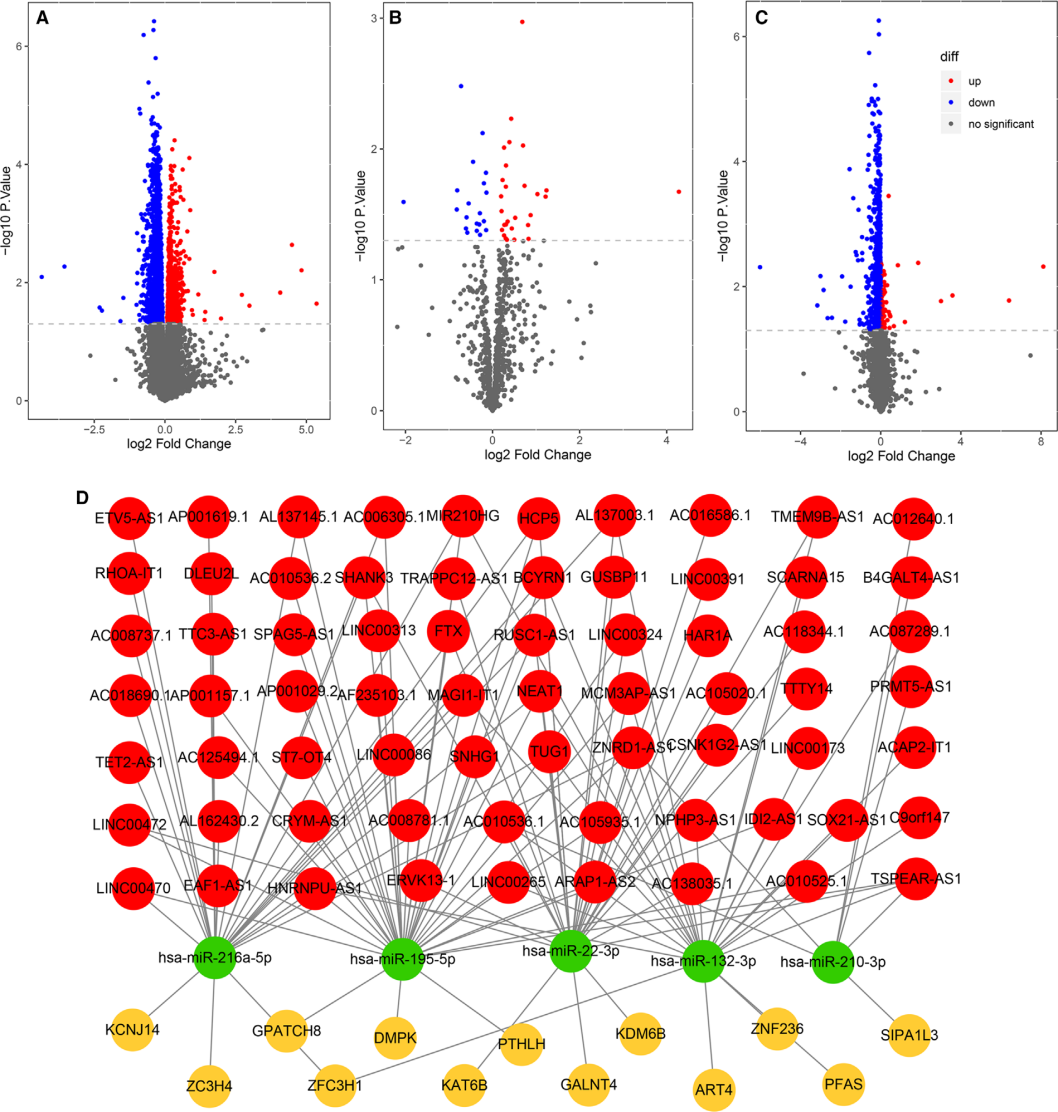
DEGs (*n* = 2899), miRNAs (*n* = 48), and lncRNAs (*n* = 1326) between reformed smokers and current smokers using the ‘limma’ package with R. (A) The DEGs. The *Y*‐axis is ‐log_10_ *P*‐value, and the *X*‐axis is log_2_Fold‐change. (B) DEGs miRNAs. (C) Differentially expressed lncRNAs. (D) The network summarizes complex connections between differentially expressed lncRNAs (red), lncRNAs targeted miRNAs (green), and DEGs (yellow).

Differentially expressed mRNA, lncRNA, and miRNA between reformed smokers and current smokers were analyzed. In total, 2899 DEGs (*P* < 0.05) were identified, including 2102 genes with significantly lower expression and 797 genes with higher expression in reformed smokers than current smokers (Fig. 1A, Table S2). GO and KEGG analysis showed that DEGs mainly enriched in the DNA and RNA‐related pathways and GO terms, including DNA replication, RNA splicing, and others (Fig. S1, Tables S3 and S4). Similarly, a total of 48 differentially expressed miRNAs (20 down‐regulated and 28 up‐regulated miRNAs, Fig. 1B) and 1326 differentially expressed lncRNAs (1207 down‐regulated and 119 up‐regulated miRNAs, Fig. 1C) were analyzed.

### 3.3 Differences in somatic mutations related to smoking status

**Fig. 2:**
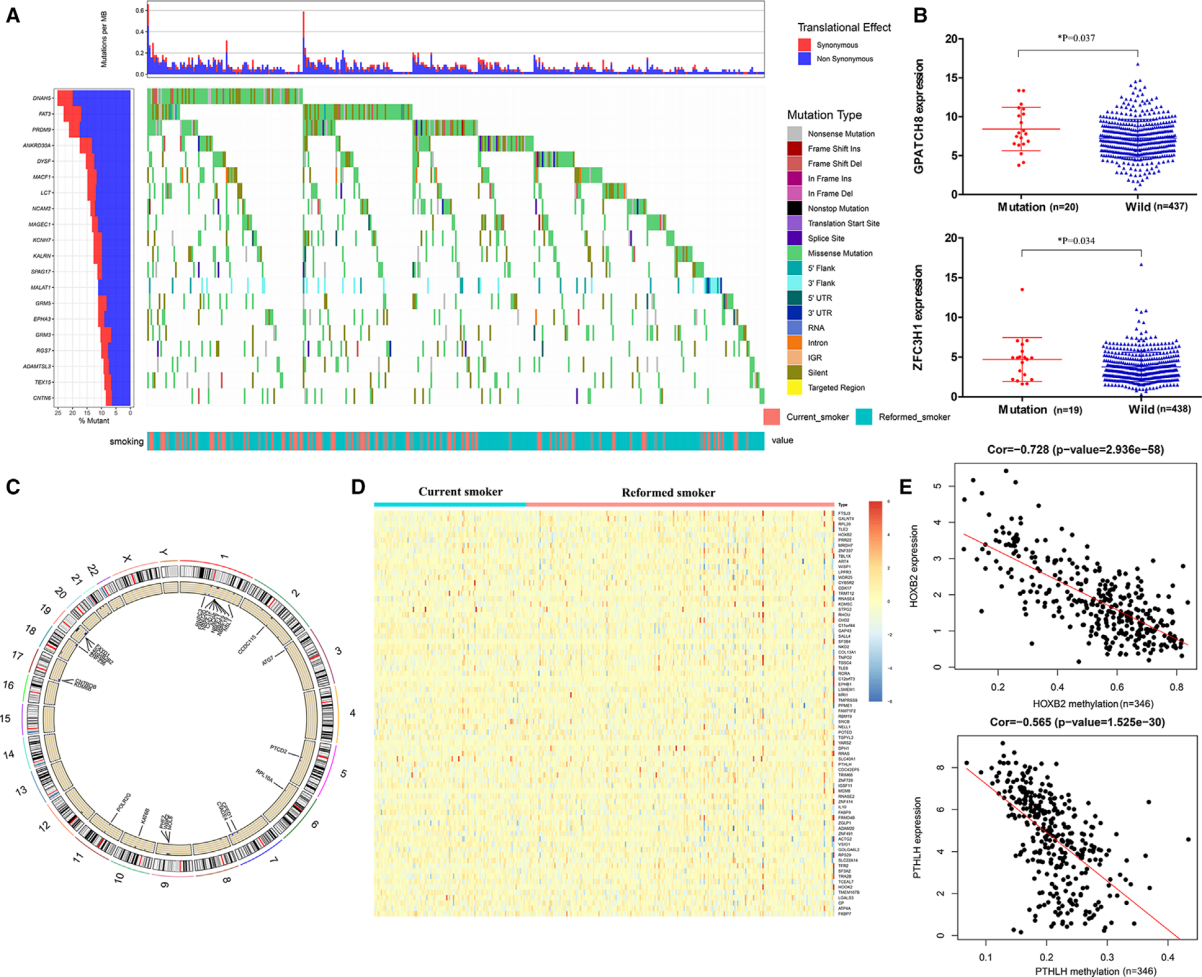
Differences in the mutational landscape and DNA methylation between reformed smokers and current smokers. (A) Top 20 differentially mutated genes between reformed smokers and current smokers. (B) The expression of GPATCH8 and ZFC3H1 was significantly associated with their somatic mutations using the Mann–Whitney *U*‐test. The error bar was SD. (C) Genes with different CNV and their copy number gains or loss mainly on chromosomes 19, 1, and 17. (D) Genes in reformed smoker with different DNA methylation compared to current smokers. (E) Expression of HOXB2 and PTHLH was significantly associated with methylation level by the Pearson correlation coefficient.

To reveal the relevant genetic alterations, we analyzed the somatic mutations between current smokers and reformed smokers. While no significant difference was found for total mutation load (Fig. S2A), relative mutations frequency of 71 genes was significantly different (Fig. 2A, Table S5) between reformed smokers and current smokers. Among them, there were 10 DEGs (Fig. S2B). We assessed whether these DEG transcriptions were affected by somatic mutations and found that the expression of GPATCH8 (*P* = 0.037) and ZFC3H1 (*P* = 0.034) was significantly associated with their somatic mutations (Figs 2B and S2C).

### 3.4 Differences in copy number variations related to smoking status

We found 781 genes with different CNV and their copy number gains or loss mainly on chromosomes 19, 1, and 17 (Fig. 2C, Table S6). Among them, we assessed whether CNVs affected transcription of 94 DEGs (Fig. S3A) and found that 73 DEGs expression was closely related to their CNVs (Table S7).

### 3.5 Differences in DNA methylation related to smoking status

To explore the impact of smoking on DNA methylation, we analyzed the gene methylation levels. We found 964 genes in reformed smokers with different DNA methylation compared to current smokers (Fig. 2D, Table S8). Among 77 DEGs (Fig. S3B), we were interested in 10 DEGs whose expression was significantly associated with methylation level (Cor < −0.30, *P* < 0.05), including HOXB2 (Cor = −0.728, *P* < 0.001) and PTHLH (Cor = −0.565, *P* < 0.001; Figs 2E and S3C).

In summary, the above analysis indicated that 85 key DEGs affected by different modes of genetic and epigenetic regulation might represent key drivers in smokers.

### 3.6 Construction of ceRNA network

Next, we constructed the ceRNA network using differentially expressed lncRNAs, miRNAs, and key DEGs. Target miRNA prediction revealed 139 lncRNA‐miRNA links, including 76 lncRNAs and eight miRNAs according to the miRcode database (Table S9). Target gene prediction for above eight miRNAs revealed 3667 miRNA‐mRNA links (prediction in at least two out of three databases) (Table S10). Based on lncRNA‐miRNA and miRNA‐mRNA links, a lncRNA‐miRNA‐DEGs complex network (69 lncRNAs, 5 miRNAs, and 13 DEGs) was built to summarize underlying molecular traits of smokers (Fig. 1D).

### 3.7 Estimation of immune cell‐type fractions in LUSC

We estimated the abundance of immune cells using three different algorithms. The distribution of several immune cell fractions in reformed smoker was different from that in current smokers, including CD8^+^ T cell (TIMER), follicular helper T cell (CIBERSORT), gamma delta T cell (CIBERSORT), M0 macrophage (CIBERSORT), central memory CD4^+^ T cell (XCELL), and central memory CD8^+^ T cell (XCELL) (Fig. S4).

### 3.8 Construction and validation of smoking signature

Univariate Cox regression analysis and LASSO‐COX analysis were performed to identify key prognostic markers, and smoking signature was built (Fig. S5, Table S11). The formula for the smoking signature was based on the corresponding coefficients (Table S12): smoking signature = 0.5410 × (smoking status) + 0.3278 × ZFC3H1|snv + 0.2153 × GPATCH8|snv + 0.3625 × NOL8|cnv + −0.5947 × RPL10A|cnv + −0.3870 × follicular helper T cell (CIBERSORT) + 0.5414 × M0 macrophage (CIBERSORT) + −0.1420 × central memory CD8^+^ T cell (XCELL).

**Fig. 3:**
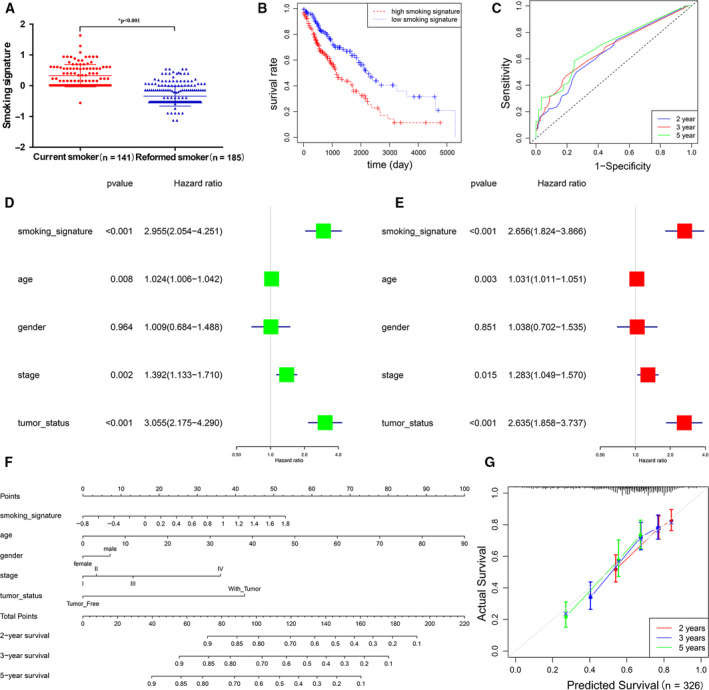
The smoking signature of evaluating the true effect of smoking on overall survival. (A) The distribution of smoking signature between reformed smokers and current smokers using Student’s *t*‐test. The error bar was SD. (B) KM curves for patients with high‐smoking signature and low‐smoking signature. (C) Survival ROC curves for 2‐, 3‐, and 5‐year prediction. (D, E) The association between smoking signature and death risk using univariate (D) and multivariate (E) Cox regression analysis. (F) Nomogram with smoking signature for predicting 2‐, 3‐, and 5‐year death risk. (G) Calibration curves of nomograms in terms of the agreement between predicted and actual 2‐, 3‐, and 5‐year outcomes. The error bar was SD.

Distributions of the smoking signature in smokers were showed that reformed smokers had lower smoking signature than current smokers (*P* < 0.001, Fig. 3A). The K‐M curves were plotted to confirm that the patients with high‐smoking signature had poorer prognosis (*P* < 0.001, Fig. 3B). The smoking signature also exhibited better‐predicted power of 2‐, 3‐, and 5‐year survival (AUC = 0.65, 0.67, and 0.70, Fig. 3C) than smoking status (AUC = 0.55, 0.55, and 0.58, Fig. S6). Moreover, univariate and multivariate Cox regression analysis showed that smoking signature could become potential independent prognostic indicators (*P* < 0.001) (Fig. 3D,E). To provide a quantitative tool to predict patients' survival, we constructed the prognostic nomogram integrating smoking signature and clinical information in smokers with LUSC (Fig. 3F). Moreover, the calibration curve of the nomogram demonstrated good agreement between prediction and observation (Fig. 3G).

To confirm the applicability and reliability of the smoking signature, we verified it in various cancers. The smoking signature of each type of cancer is provided in Table S13. In age‐adjusted models, the smoking signature was significantly associated with overall survival in BLCA, CESC, HNSC, LUAD, LUSC, and PAAD. In multivariable‐adjusted model, patients with higher smoking signature had higher hazard rates than patients with lower smoking signature in BLCA (HR = 1.70, 95% CI = 1.01–2.88), CESC (HR = 5.69, 95% CI = 1.37–23.69), HNSC (HR = 1.97, 95% CI = 1.41–2.76), LUAD (HR = 1.73, 95% CI = 1.16–2.57), LUSC (HR = 1.70, 95% CI = 1.19–2.43), and PAAD (HR = 4.28, 95% CI = 1.47–12.47) (Table 2). We also constructed the prognostic nomogram with the smoking signature in BLCA, CESC, ESCA, HNSC, LUAD, LUSC, and PAAD as quantitative tools (Fig. S7).

**Table 2:** The association between the smoking signature and patients' overall survival^a^.

Cancer type	Age‐adjusted HR (95% CI)	*P*‐value	MV‐adjusted HR (95% CI)^b^	*P*‐value
BLCA	**1.03 (1.01**–**1.05)**	**0.001**	**1.70 (1.01**–**2.88)**	**0.048**
CESC^c^	**4.69 (1.80**–**12.23)**	**0.002**	**5.69 (1.37**–**23.69)**	**0.017**
ESCA	1.22 (0.50–3.01)	0.663	1.30 (0.50–3.41)	0.596
HNSC^c^	**2.14 (1.55**–**2.96)**	**< 0.001**	**1.97 (1.41**–**2.76)**	**< 0.001**
LUAD	**1.52 (1.07**–**2.17)**	**0.020**	**1.73 (1.16**–**2.57)**	**0.007**
LUSC	**1.85 (1.29**–**2.64)**	**0.001**	**1.70 (1.19**–**2.43)**	**0.003**
PAAD	**5.90 (2.60**–**13.41)**	**< 0.001**	**4.28 (1.47**–**12.47)**	**0.008**

Significant associations are shown in bold.

^a^ The smoking signature is the continuous variable.

^b^ MV‐adjusted for age (continuous), gender (female, male), tumor stage (stage Ⅰ, stage Ⅱ, stage Ⅲ, and stage Ⅳ), and tumor status (with tumor and tumor‐free).

^c^ MV‐adjusted for age (continuous), gender (female, male), tumor stage (stage Ⅰ, stage Ⅱ, stage Ⅲ, and stage Ⅳ), tumor status (with tumor and tumor‐free), and HPV status (positive and negative).

## 4 Discussion

Tobacco smoking is an established risk factor for many cancers' development. It is known that smoking cessation reduces mortality and increases the life span. However, a causal relationship between smoking cessation and prognosis in cancer patients who are current smokers at the time of a cancer diagnosis is still unclear [23], and the lack of a special tobacco smoking assessment signature potentially underestimates the true impact of smoking on overall survival. In the present study, we used the TCGA cohort to estimate the association between smoking cessation and overall survival, understand the genetic and immune microenvironment of smoking patients, and provide an effective smoking signature for evaluating the smoking level to predict prognosis.

By performing age‐adjusted Cox regressions, we found that smoking at diagnosis increased mortality risk as compared with reformed smokers in CESC, HNSC, LUSC, and PAAD. Importantly, quitting smoking was the independent prognostic factor of LUSC. Several studies have evaluated the effect of smoking on LUSC. Molinier *et al*. [24] estimated 5‐year survival in non‐small‐cell lung cancer patients and found that smoking level at diagnosis was an independent negative prognostic factor in LUSC patients. Nakamura *et al*. [25] performed the multivariate analysis to find that smoking in LUSC was associated with recurrence‐free survival. Although without distinguishing histological type, a cohort claimed that recent quitting could decrease the risk of death among patients with lung cancer (HR, 0.90; 95% CI 0.81–0.99) [11]. Synthesizing above all outcomes, it is demonstrated reducing smoking could decrease the deterioration risk as compared with current smoking, suggesting a reversible effect of smoking in LUSC.

Then, we analyzed the somatic mutation, CNV, and DNA methylation between reformed smokers and current smokers. A total of 85 key DEGs were identified, whose expression was regulated by gene mutation or methylation. Among them, several key genes have been confirmed to be associated with LUSC. CBLC can be recruited into the epidermal growth factor receptor (EGFR) to increase EGFR ubiquitination, and thereby downregulate EGFR signaling in lung cancer patients [26]. Zhan *et al*. [27] suggested that RPS11 was considered as the suitable reference gene for qRT‐PCR‐based studies of squamous cell lung carcinoma because of its high and stable expression. Sienel *et al*. [28] found that CEACAM1 has implicated in the development and progression of LUSC and an independent prognosticator for survival. Besides, these key genes also played important roles in smoking‐related cancers, including lung cancer. Shui *et al*. [29] found the DNA methylation of LGALS3 was associated with smoking status in prostate cancer and strongly correlated with its expression. DNA ligase I (LIG1) is a DNA repair gene involved in both the nucleotide excision repair and the base excision repair pathways [30]. Many studies confirmed that variants in LIG1 may predispose to smoking‐related lung cancer [31, 32]. By comparing the gene expression profiles in lung cancer between nonsmokers and smokers, Woenckhaus *et al*. [33] found PTHLH, being involved in matrix degradation, was differentially expressed, which could reflect early cigarette smoke‐induced and cancer‐relevant molecular lesions. Chronic obstructive pulmonary disease (COPD) is another threat of smoking‐induced lung injury, which can be the driving factor for lung cancer [34]. ATG7, an autophagic gene, is increasingly activated in the early stages of lung injury induced by cigarette smoke [35, 36]. AXL is a receptor tyrosine kinase related to cancer and immune function, which mediates signal transduction related to proliferation and inflammation [37]. During secondhand smoke, the interaction between AXL and receptors for advanced glycation end products can cause COPD. Exposure to cigarette smoke, LGALS3 can increase CXCL8 secretion to induce inflammation [38] in COPD. Nowadays, the importance of smoking cessation in the management of COPD has been well‐established [39]. Similarly, cancer patients should quit smoking as soon as possible, which is helpful for cancer treatment by regulating key genes [40]. In addition, we constructed the ceRNA network to summarize the underlying molecular traits of smokers, indicating that smoking could affect DEGs by different modes of genetic and epigenetic regulation.

Therefore, we provided a comprehensive smoking signature including immune microenvironment and epigenetic regulation to evaluate the true impact of smoking because of the complexity in cancer smokers. By understanding the immune microenvironment, we found that the fractions of follicular helper T cell, M0 macrophage, and central memory CD8+ T cell were different between reformed smokers and current smokers, suggesting that smoking status could change immune microenvironment to affect prognosis. It is reported that immune homeostasis in tumor microenvironment appears to be less compromised in nonsmokers than in ever‐smokers. In addition, the composition of leukocyte subtypes is closely correlated not only with smoking history, but also with patients' outcome [41]. Different subsets of T cells are playing different roles in immune response [42]. Follicular helper T cells play crucial roles in the development of humoral immunity [43]. Yang *et al*. [13] estimated the immune cell‐type fractions in digestive system cancer and found that follicular helper T cells were the protective factors of patients' overall survival. Many studies also have shown that CD8+ T cells usually mean a better prognosis among cancer patients [44, 45]. M0 macrophages were reported to be inversely associated with patients' outcomes in various, such as adrenal cortical carcinoma and lung cancer [46, 47]. Nowadays, majority studies evaluated the degree of smoking based on the frequency of tobacco use to define heavy smokers and light smokers without uniform quantifying standards [48, 49, 50]. Moreover, these cut‐offs cannot accurately identify the true degree of smoking because they do not comprehensively consider the DNA damage and microenvironment alteration. Rosenthal *et al*. [51] developed deconstructSigs to identify mutational smoking signature in LUSC, LUAD, and HNSC. Desrichard *et al*. confirmed that patients with high mutational smoking signature had poorer overall survival in HNSC (HR = 1.50, 95% CI = 1.23–1.81, *P* < 0.01), but the mutational smoking signature was not prognostic in LUSC (HR = 1.02, 95% CI = 0.71–1.46, *P* = 0.92) [52] and LUAD (HR = 1.18, 95% CI = 0.46–3.04, *P* = 0.74). Importantly, the smoking signature we constructed not only can predict the overall survival in LUSC, but also can serve as prognostic indicators in BLCA, CESC, HNSC, LUAD, and PAAD.

Nevertheless, the present study is not without limitations. First, information on the timing of smoking cessation is lacking, which cannot tell whether quitting earlier is better for overall survival. Second, smoking status after diagnosis is unknown—it is possible that some patients relapsed to smoking after diagnosis, which could have biased the results. In the future, we need large cohorts with complete smoking information for further study.

## 5 Conclusion

The present study demonstrated that quitting smoking at diagnosis decreased the risk of death in cancer patients, suggesting a reversible effect of smoking on prognosis. We further provided the smoking signature by understanding the underlying molecular traits to evaluate the true effect of smoking, which could improve the prognostic prediction. At the same time, we suggested that smoking cessation could be a part of cancer treatment to improve the survival rate of cancer patients.

## Conflict of interest

The authors declare no conflict of interest.

## Author contributions

YS and GY conceived and designed the project; YS and LT acquired the data; YS and LT analyzed and interpreted the data; and YS wrote the paper.

## Supporting information


**Fig. S1.** The GO and KEGG analysis of DEGs.
**Fig. S2.** Differences in somatic mutations related to smoking status.
**Fig. S3.** Differences in DNA methylation related to smoking status.
**Fig. S4.** Understanding the immune microenvironment using three algorithms.
**Fig. S5.** Smoking signature built using the LASSO model.
**Fig. S6.** Survival receiver–operating characteristic (ROC) curves of smoking status for 2,3, 5‐year prediction.
**Fig. S7.** Clinical application of nomograms with smoking signature in smoking‐related cancers.

**Table S1**. Detailed patient characteristics of each cancer in TCGA.

**Table S2**. Differentially expressed genes (DEGs) between reformed smokers and current smokers.
**Table S3**. GO analysis.
**Table S4**. KEGG analysis.
**Table S5**. Relative mutations frequency of 71 genes was significantly different.
**Table S6**. 781 genes were with different CNV.
**Table S7**. 73 DEGs expression was closely related to their copy number variations.
**Table S8**. 964 genes in reformed smokers with different DNA methylation compared to current smokers.
**Table S9**. The lncRNA‐miRNA links.
**Table S10**. The miRNA‐mRNA links.
**Table S11**. The univariate Cox regression analysis.
**Table S12**. The corresponding coefficients in LASSO‐COX analysis.
**Table S13**. The smoking signature of each type of cancer in TCGA.

## Data Availability

The datasets generated during and/or analyzed during the current study are available from the corresponding author on reasonable request. The data that support the findings of this study were derived from the following resource: TCGA database, https://www.cancer.gov/tcga.
