# Supplementary material for: The benefits of smoking cessation on survival in cancer patients by integrative analysis of multi‐omics data
Source: Mol Oncol. 2020 Jul 11;14(9):2069–80. doi: 10.1002/1878-0261.12755 (PMC7463331; doi:10.1002/1878-0261.12755)

Supplementary Figure 1: the GO and KEGG analysis of DEGs. A: GO analysis. B: KEGG analysis

A

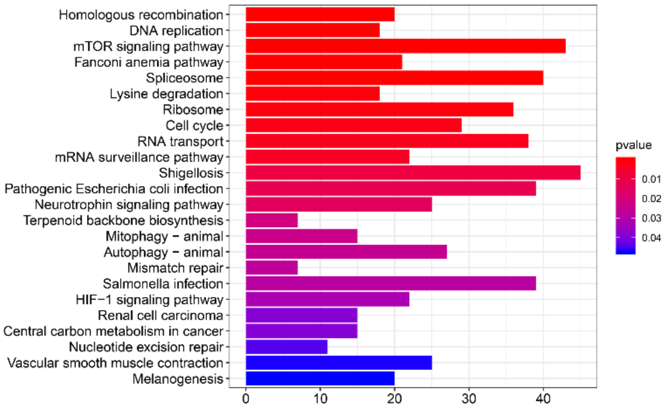

B

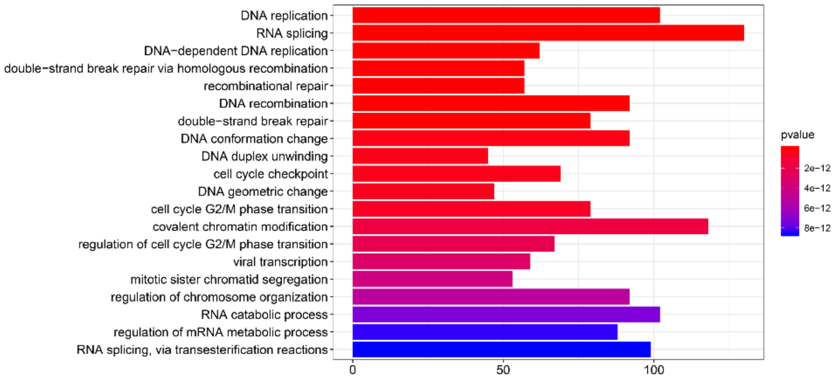

Supplementary Figure 2: Differences in somatic mutations related to smoking status. A: total mutation load between reformed smokers and current smokers. B: mutations frequency of ten DEGs was significantly different between reformed smokers and current smokers. C: the association between somatic mutations and expression of ten DEGs.

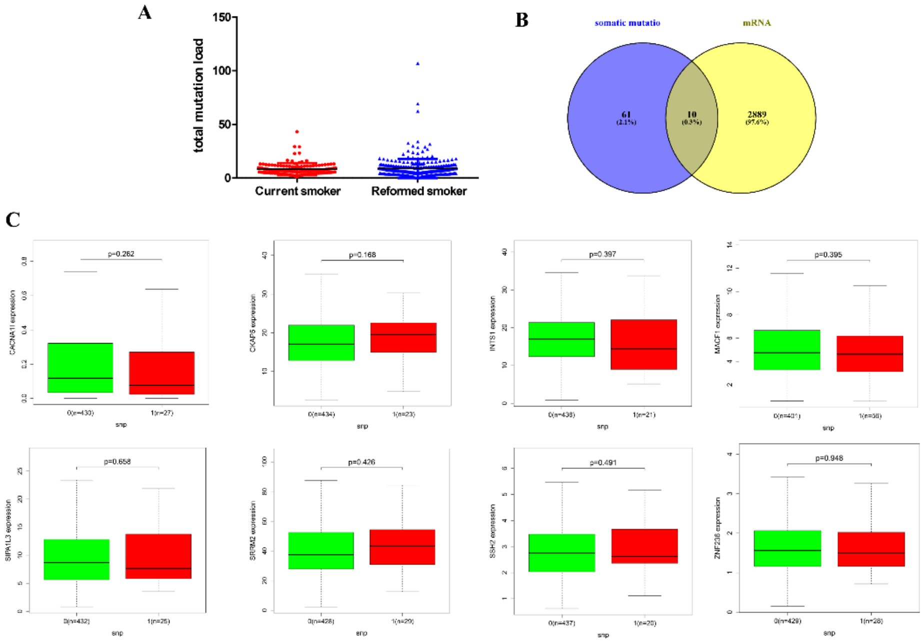

Supplementary Figure 3: Differences in DNA methylation related to smoking status. A: 94 DEGs with different CNV between reformed smokers and current smokers. B: 77 DEGs with different DNA methylation. C: expression of ten DEGs were significantly associated with methylation level.

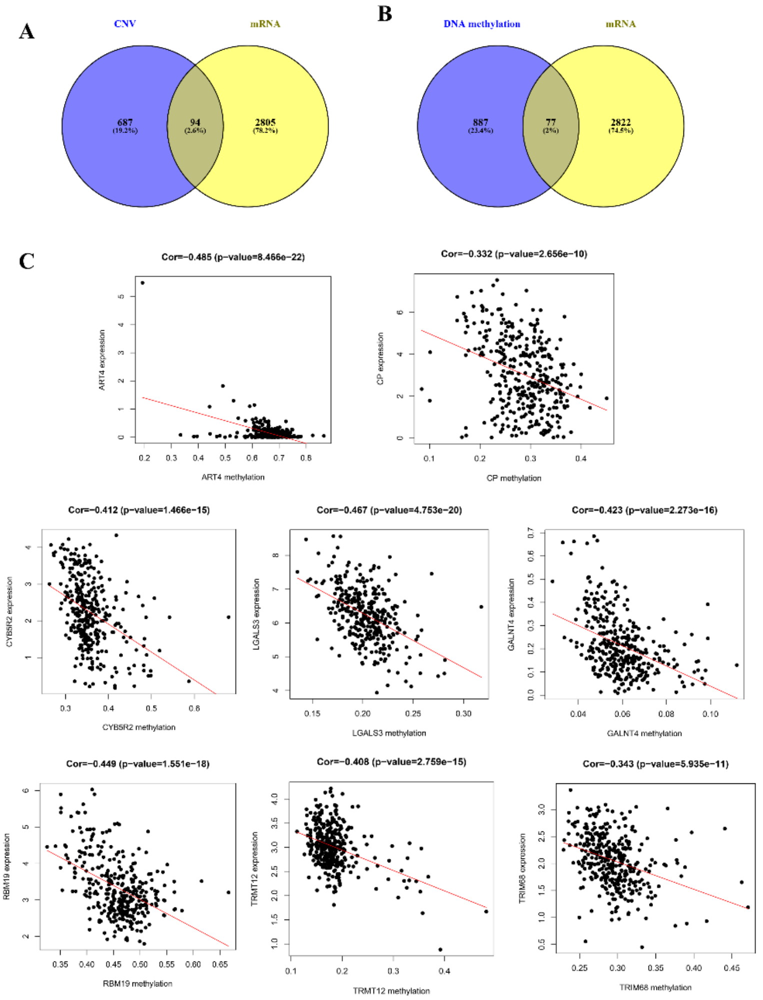

Supplementary Figure 4: Understanding the immune microenvironment using three algorithms. A: TIMER. B: CIBERSORT. C: XCELL.

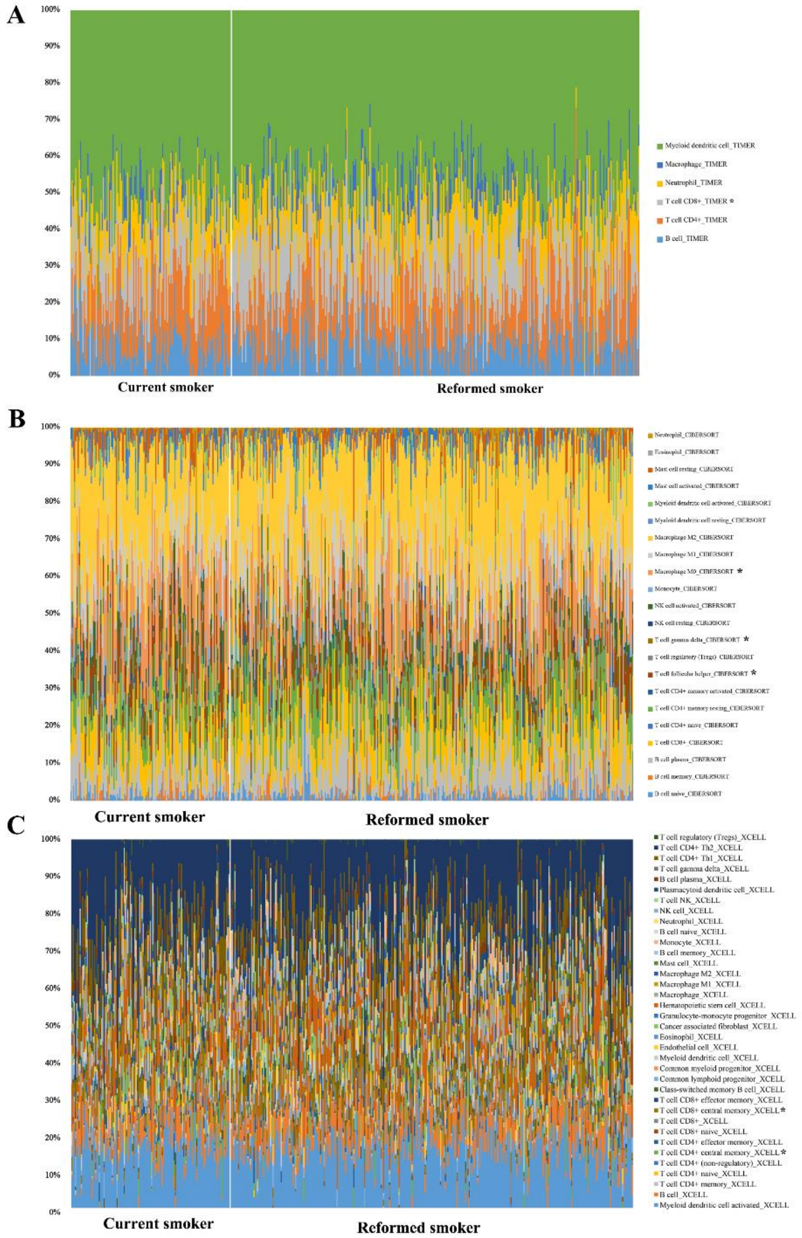

Supplementary Figure 5: Smoking signature built using the LASSO model. A: LASSO coefficient profiles of different factors. B: Tenfold cross-validation for tuning parameter selection in the LASSO model.

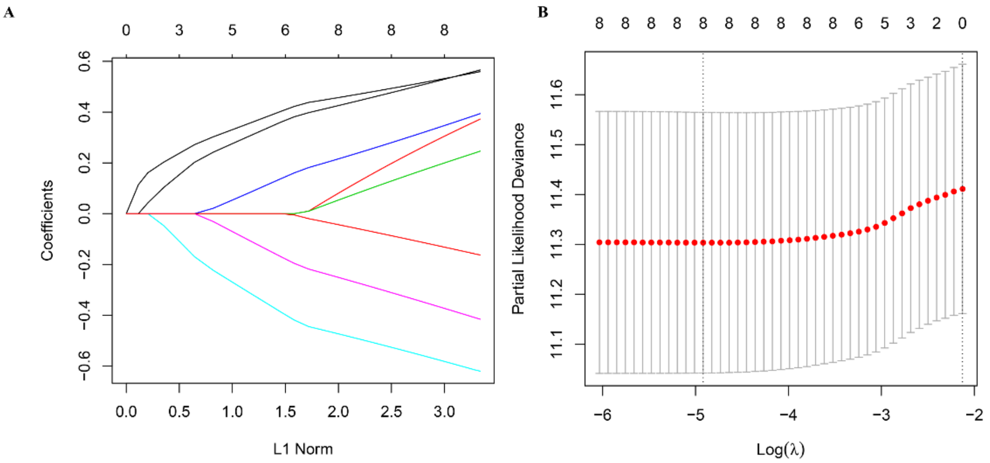

Supplementary Figure 6: Survival receiver–operating characteristic (ROC) curves of smoking status for 2,3, 5-year prediction.

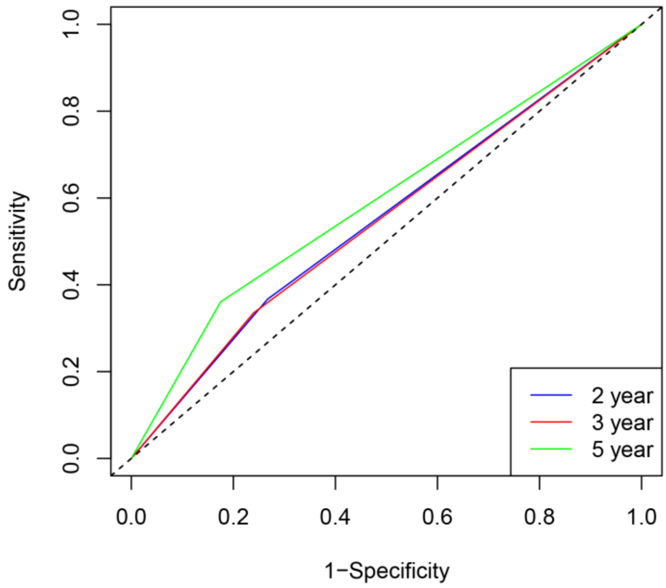

Supplementary Figure 7: Clinical application of nomograms with smoking signature in smoking-related cancers. A: BLAC. B: CESC. C: ESCA. D: HNSC. E: LUAD. F: PAAD.

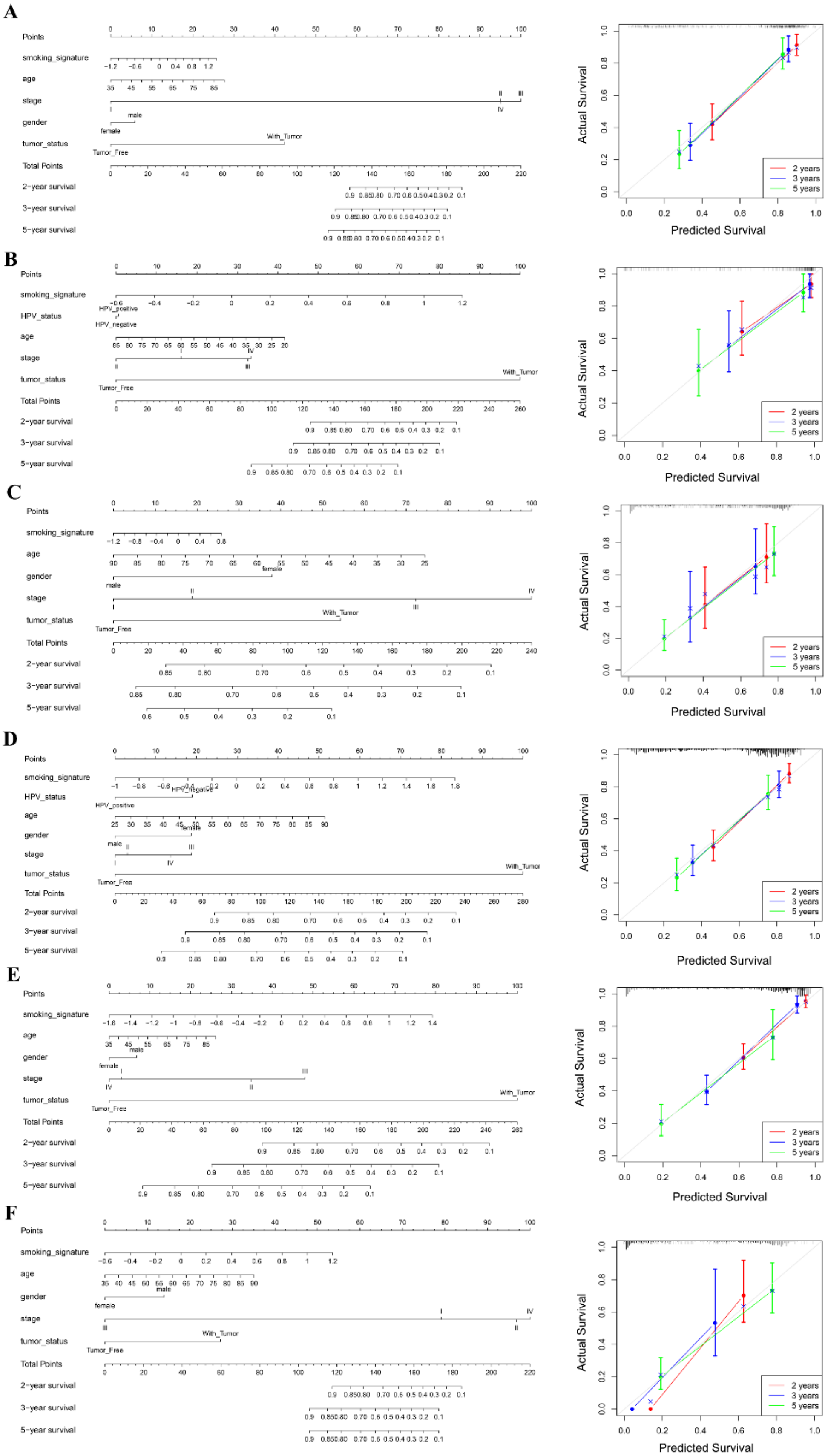

Supplement: Supplementary file 1 — Fig. S1. The GO and KEGG analysis of DEGs. Fig. S2. Differences in somatic mutations related to smoking status. Fig. S3. Differences in DNA methylation related to smoking status. Fig. S4. Understanding the immune microenvironment using three algorithms. Fig. S5. Smoking signature built using the LASSO model. Fig. S6. Survival receiver–operating characteristic (ROC) curves of smoking status for 2,3, 5‐year prediction. Fig. S7. Clinical application of nomograms with smoking signature in smoking‐related cancers. [file MOL2-14-2069-s001.pdf]
